# Intraarterial transplantation of human umbilical cord blood mononuclear cells in hyperacute stroke improves vascular function

**DOI:** 10.1186/s13287-017-0529-y

**Published:** 2017-03-22

**Authors:** Lei Huang, Yichu Liu, Jianfei Lu, Bianca Cerqueira, Vivek Misra, Timothy Q. Duong

**Affiliations:** 1 Research Imaging Institute, University of Texas Health Science Center, San Antonio, Texas USA; 2 Department of Biomedical Engineering, University of Texas, San Antonio, Texas USA; 3 Department of Neurology, University of Texas Health Science Center, San Antonio, Texas USA; 4 Radiology, Stony Brook Medicine, Stony Brook, NY USA

**Keywords:** Umbilical cord blood cell, Stroke, Cell transplantation, CBF, MRI, Vasoreactivity

## Abstract

**Background:**

Human umbilical cord blood (hUCB) cell therapy is a promising treatment for ischemic stroke. The effects of hyperacute stem cell transplantation on cerebrovascular function in ischemic stroke are, however, not well understood. This study evaluated the effects of hyperacute intraarterial transplantation of hUCB mononuclear cells (MNCs) on cerebrovascular function in stroke rats using serial magnetic resonance imaging (MRI).

**Methods:**

HUCB MNCs or vehicle were administered to stroke rats via the internal carotid artery immediately after reperfusion at 60 min following ischemia onset. Lesion volumes were longitudinally evaluated by MRI on days 0, 2, 14, and 28 after stroke, accompanied by behavioral tests. Cerebral blood flow (CBF) and cerebrovascular reactivity were measured by perfusion MRI and CO_2_ functional MRI (fMRI) at 28 days post-stroke; corresponding vascular morphological changes were also detected by immunohistology in the same animals.

**Results:**

We found that CBF to the stroke-affected region at 28 days was improved (normalized CBF value: 1.41 ± 0.30 versus 0.49 ± 0.07) by intraarterial transplantation of hUCB MNCs in the hyperacute stroke phase, compared to vehicle control. Cerebrovascular reactivity within the stroke-affected area, measured by CBF fMRI, was also increased (35.2 ± 3.5% versus 12.8 ± 4.3%), as well as the corresponding cerebrovascular density. Some engrafted cells appeared with microvascular-like morphology and stained positive for von Willebrand Factor (an endothelial cell marker), suggesting they differentiated into endothelial cells. Some engrafted cells also connected to host endothelial cells, suggesting they interacted with the host vasculature. Compared to the vehicle group, infarct volume at 28 days in the stem cell treated group was significantly smaller (160.9 ± 15.7 versus 231.2 ± 16.0 mm^3^); behavioral deficits were also markedly reduced by stem cell treatment at day 28 (19.5 ± 1.0% versus 30.7 ± 4.7% on the foot fault test; 68.2 ± 4.6% versus 86.6 ± 5.8% on the cylinder test). More tissue within initial perfusion-diffusion mismatch was rescued in the treatment group.

**Conclusions:**

Intraarterial hUCB MNC transplantation during the hyperacute phase of ischemic stroke improved cerebrovascular function and reduced behavioral deficits and infarct volume.

## Background

Cell therapy is a promising treatment for ischemic stroke. Several preclinical stroke studies have shown beneficial effects following transplantation of stem cells derived from various sources, including embryonic tissue [1], adult bone marrow [2], adipose [3], and umbilical cord blood (UCB) [4, 5]. Amongst these, UCB cells offer several advantages because they are more readily available and have no associated graft-versus-host reactions [6]. Unlike cells derived from embryonic sources, there are no ethical issues with using cells derived from UCB. UCB cells have been used clinically to treat hematological malignancies for more than two decades with a good safety record [7]. Transplantation of human UCB (hUCB) mononuclear cells (MNCs) and their different subcomponents (i.e., hematopoietic stem cells (HSCs), mesenchymal stem cell (MSCs), and endothelial progenitor cells (EPCs)) have also been shown to be effective in animal models of ischemic stroke [4, 5, 8].

Most preclinical studies evaluated cell administration 24 h or later after stroke, and reported outcomes using behavioral assessment and histology, and a few used T_2_-weighted magnetic resonance imaging (MRI) to measure infarct volume [4, 5, 9, 10]. In addition to quantifying lesion volume and the tissue at-risk (i.e., “perfusion-diffusion” mismatch) [11, 12] in a longitudinal manner, MRI can also be used to measure cerebral blood flow (CBF) and CBF responses to physiological and functional challenges, providing a noninvasive method to study neuron-vascular coupling and hemodynamic regulation after stroke and during recovery [12]. CBF and cerebrovascular reactivity are known to be perturbed after stroke [12–14], and recovery of CBF and cerebrovascular reactivity plays an important role in functional recovery in ischemic stroke [15]. However, the effects of hUCB MNC transplantation on vascular function in vivo (i.e., CBF, cerebrovascular reactivity, and the underlying vascular changes) are not well understood.

The goal of this study was to evaluate the effects of hyperacute intraarterial transplantation of hUCB MNCs on cerebral vascular function in a rat model of focal cerebral ischemia. We hypothesize that such treatment improves CBF and cerebrovascular reactivity on the stroke-affected region, with reducing behavioral deficits and infarct volume. The intraarterial delivery method was intended to mimic the clinical condition in which stem cell treatment could be administered following mechanical thrombectomy where a catheter is already in place [16, 17]. The effects of intraarterial stem cell infusion on the ischemic lesion volume were longitudinally evaluated by MRI on days 0, 2, 14, and 28 days following stroke, accompanied with behavioral tests. CBF and cerebrovascular reactivity were measured by perfusion MRI and CO_2_ fMRI at 28 days post-stroke; corresponding vascular morphological changes were also detected by immunohistology in the same animals.

## Methods

All experiments followed guideline and regulations consistent with the Guide for the Care and Use of Laboratory Animals, Public Health Service Policy on Humane Care and Use of Laboratory Animals, and the Animal Welfare Act and Animal Welfare Regulations. Animal experiments were approved by the Institutional Animal Care and Use Committee of the University of Texas Health Science Center San Antonio. Animals arrived at our facility at least 5 days before experimentation. Rats were housed two per cage prior to stroke and one per cage after stroke in a Tecniplast caging system with autoclaved Sani-chip bedding with 12-h light and 12-h dark cycle. Rats had ad libitum access to irradiated rodent chow from Harlan laboratories and autoclaved water. In addition, gel food (Bio-Serv) was provided in the cage after stroke. Buprenex (0.05 mg/kg) was injected subcutaneously for the first 3 days after surgery.

Sample sizes were calculated via Lamorte’s Power calculation [18] (University of Boston) with α = 0.05. Expected variances and differences between groups were derived from pilot experiments or the reports of others [5]. For infarction evaluation, effect size = 1.78, six animals per group were needed to achieve >80% power. For behavior experiments, effect size =1.95, six animals per group were needed to achieve >80% power. Experiments and analysis were performed in a blinded manner.

### Middle cerebral arterial occlusion model and experiment groups

Twenty male Sprague Dawley rats (250–320 g; Charles River Laboratories, USA) were subjected to transient (60 min) middle cerebral arterial occlusion (MCAO) via intraluminal vascular occlusion, as previously described [19, 20]. Rats were anesthetized initially with 3.5% isoflurane mixed with room air and maintained at 2.0% isoflurane during surgery, and 1.5% during MRI. The left common carotid artery and external carotid artery (ECA) were exposed through a midline neck incision. A silicon rubber-coated filament (Doccol Corporation, Sharon, MA, USA) was introduced into the left internal carotid artery (ICA) through the ECA stump to occlude the origin of the MCA. After a 60-min occlusion, reperfusion was achieved by filament withdrawal. The rectal temperature was maintained at 37 ± 0.5 °C. The heart rate and blood oxygen saturation level were monitored using a MouseOx system (STARR Life Science Corp., Oakmont, PA, USA). All recorded physiological parameters were maintained within normal physiological ranges (arterial O_2_ saturation 91–98%, heart rate 350–410 bpm).

**Fig. 1:**
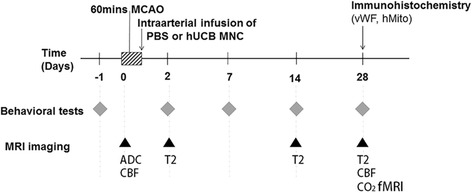
Timeline of the experiment. Phosphate-buffered saline (*PBS*) or human umbilical cord blood (*hUCB*) mononuclear cells (*MNCs*) were transplanted immediately after 60-min middle cerebral arterial occlusion (*MCAO*). Behavioral tests and magnetic resonance imaging (*MRI*) were performed at the indicated time points. *ADC* apparent diffusion coefficient, *CBF* cerebral blood flow, *fMRI* functional MRI, *hMito* human mitochondria, *vWF* von Willebrand factor

Stroke rats were randomly assigned to two groups: (1) phosphate-buffered saline (PBS) as a vehicle control; or (2) hUCB MNCs via intraarterial transplantation after reperfusion. In order to minimize the variability of MCAO, the exclusion criteria were that initial apparent diffusion coefficient (ADC) lesion volume at 30 min after MCAO was <100 mm^3^, indicative of incomplete occlusion. One rat from each group died during the follow-up study; the mortality rate (1/10) was equal in the two groups. Three rats from each group were excluded due to small initial ADC lesion. The final sample sizes were six rats for each group. The experimental design is shown in Fig. 1.

### Cell preparation and transplantation

Intracarotid cell transplantation was performed immediately after reperfusion. Cryopreserved hUCB MNCs were purchased from StemCell Technologies (#70007; Vancouver, Canada) which were separated from the cord blood of a healthy donor by density gradient centrifugation. Cells were rapidly thawed at 37 °C and passed through a sterilized 70-μm filter (Thermo Fisher). The cell count of a single cell suspension and viability was quantified by the trypan-blue dye exclusion method. The volume was adjusted for a total amount of 5 × 10^6^ hUCB MNCs in 35 μl PBS. Immediately following withdrawal of the filament, the ipsilateral ECA stump was cannulated with a PE-5 tube which was connected to a 50-μl microneedle Hamilton syringe filled with cell suspension. The distal end of the PE-5 tube was navigated to the extracranial part of the ICA. The pterygopalatine artery was temporarily ligated. A cell suspension of 35 μl was infused over the course of 5 min into the ICA. Rats in the vehicle group were infused with the same volume of PBS.

### MRI

MRI was performed on a Bruker 7-T BioSpec Scanner with a 40 G/cm BGA12S gradient insert (Billerica, MA, USA). A custom-made surface coil (2.3-cm internal diameter) and a neck coil were used for brain imaging and perfusion labeling separately. Rectal temperature was monitored and maintained at 37.0 ± 0.5 °C during the MRI scan using a thermostatically controlled water flow system. MRI was acquired at 30 min after MCAO and after reperfusion, and again at 2, 14, and 28 days after MCAO.

#### ADC

Diffusion-weighted images were acquired using a single-shot, spin-echo, echo-planar imaging (EPI) sequence, with the following parameters: matrix = 96 × 96, reconstructed to 128 × 128, FOV = 2.56 × 2.56 cm, seven 1.5-mm slices, TR = 3 s, and TE = 37 ms. Two levels of diffusion sensitization (b = 0 and 1200 s/mm^2^), applied along the x, y, z direction separately, were used to calculate the ADC map [19]. Total acquisition time = 3.5 min.

#### CBF

The continuous arterial spin-labeling (cASL) technique was performed to measure CBF as previously described [19, 20]. cASL employed a 2.7-s square radiofrequency pulse to the labeling coil. A single-shot, gradient-echo, EPI sequence was used with the following parameters: matrix = 96 × 96, reconstructed to 128 × 128, FOV = 2.56 × 2.56 cm, seven 1.5-mm slices, TR = 3 s, flip angle = 90°, and TE = 14 ms. Pair images with and without tagging were acquired. Total acquisition time = 6 min. For CBF, 60 repetitions were obtained and averaged. To investigate the response to hypercapnic challenge at 28 days after stroke, dynamic CBF was acquired for 2 min during air inhalation, then for 3 min during 5% CO_2_ (premixed) inhalation, and subsequently 5 min of inhalation of air [21].

#### T2

T2-weighted image was acquired using fast spin-echo sequence, with TR = 3 s and four effective TE (25, 40, 75, and 120 ms) to generate T2 maps. Other parameters were: seven 1.5-mm coronal images, FOV = 2.56 × 2.56 cm, matrix 96 × 96, reconstructed to 128 × 128, and 8 transients for signal averaging. Total acquisition time = 8 min.

### Imaging analysis

ADC, CBF, T2 map and CO_2_ reactivity maps were generated and analyzed using Matlab (MathWorks Inc., Natick, MA, USA) and STIMULATE (University of Minnesota) as previously described [19, 22]. Image maps of individual subjects were co-registered across time points by QuickVol and MRIAnalysisPak software. Stroke-induced initial lesion was defined by an abnormal ADC 30 min post-MCAO with an established threshold (0.53 × 10^–3^ mm^2^/s) [23]. The ischemic core and perfusion-diffusion mismatch were defined based on 30-min ADC and CBF maps using previously described measurements [12, 19]. The fate of the initial ischemic core and mismatch tissues were tracked over time. Lesion volume was calculated based on the T2 map at 2 days post-MCAO due to a better resolution than the ADC map. The lesion area was defined by the pixels with T2 value higher than the mean value plus two times the standard deviation (mean + 2SD) provided by the contralateral side of the brain. Lesion volume was quantified by summing all the lesion areas measured on all slices and multiplying by the slice thickness. Edema correction was performed for lesion calculation of day 2 data as reported previously [19]. The normal CBF value was defined as a range of values two standard deviations above and below the mean (mean ± 2SD) obtained from the contralateral side. Three regions of interest (ROIs) were generated to separate normal, hyper- and hypoperfusion, based on CBF values. For CO_2_ reactivity, CBF percentage change maps were calculated by modeling the time course to the input hypercapnic paradigm using STIMULATE Software, as previously described [22, 24]. Investigators performing image analysis were blinded to the experimental groups.

### Histology

Immunohistology for von Willebrand Factor (vWF) was performed immediately after MRI experiments on day 28 post-MCAO. Briefly, rats were anesthetized and perfused transcardially with 4% paraformaldehyde (PFA). Brains were removed and postfixed in 4% PFA for 24 h at 4 °C and subsequently cryopreserved in 30% sucrose for 2 days. Brain samples were frozen in 20-μm sections and mounted on gelatin-coated slides. Immunofluorescent staining was performed on a separate section correlating with MRI slices. Briefly, frozen sections were blocked by incubation in 10% goat serum in PBS after washing with PBS (PH 7.4, containing 0.1% Tween 20, 0.3% Triton X-100). Sections were then incubated with primary antibodies at 4 °C overnight. Primary antibodies used were rabbit anti-vWF (1:100; Abcam #ab6994, Cambridge, MA, USA) and mouse anti-human mitochondria (1:50; Millipore #MAB1273, Temecula, CA, USA). After washing with PBS, the slides were then incubated with the secondary antibodies goat anti-rabbit Alexa Fluor 488 (1:300) and goat anti-mouse Alexa Fluor 594 (1:300; Invitrogen, USA). Slides were washed and mounted with mounting solution containing 4’,6’-diamidino-2-phenylindole (DAPI; Vector Laboratories, H-1400). Images were acquired with a Zeiss laser scanning microscope (LSM710) and double-labeling confirmed using z-stacks scan. For each brain sample, two sections with a 0.2-mm gap, correlated to the same level as the MRI image, were used for vWF staining and quantification. On each section, three ROIs (within normal, hyper-, and hypoperfusion) were selected based on MRI CBF data. Digital images were captured on four randomly selected fields within each ROI. The mean fluorescent intensity of vWF staining was quantified as previously described [25] using Zen2012. Data from all six samples were averaged and compared between the two groups.

### Functional assessment

The foot-fault test and cylinder test were performed to evaluate the sensorimotor function of the rats at 1 day before surgery and 2, 7, 14, and 28 days post-MCAO. The foot-fault test measures the forelimb misplacement on a grid during locomotion. The performance of the rats was videotaped for 5 min or until 50 steps were taken with one forelimb (non-affected). The total number of steps and number of times each forelimb fell below the grid were counted by an observer blinded to the experimental groups. The percentage of foot-faults for the right forelimb (affected by the stroke) to total steps was calculated and presented as previous reported [26].

The cylinder test were performed to determine the asymmetrical use of the forearm. Animal were video recorded in a transparent cylinder (20-cm diameter by 30-cm height) for 5 min. Forelimb placement on the wall was counted by an observer blinded to the experimental groups. The frequency of left forelimb (unaffected) placement to total placement was calculated and expressed as previous described [26].

### Statistical analysis

A two-way analysis of variance (ANOVA; animal group and different ROIs) with Bonferroni post-hoc test was used to compare behavioral scores, lesion volumes, the percentage of pixels with normal perfusion, hyperperfusion, and hypoperfusion on regions corresponding to T_2_ abnormal maps, CO_2_-induced CBF percentage changes in normal perfusion, hyperperfusion, and hypoperfusion regions, and vWF fluorescent density in normal perfusion, hyperperfusion, and hypoperfusion regions between the two animal groups. The unpaired two-tailed *t* test was used to compare percentage of tissue rescued, CBF values, and CO_2_-induced CBF percentage changes between the vehicle and treatment group. Values are expressed as mean ± standard error of the mean (SEM). *P* values <0.05 were taken as statistically significant.

## Results

### hUCB MNC transplantation significantly improved functional recovery

**Fig. 2:**
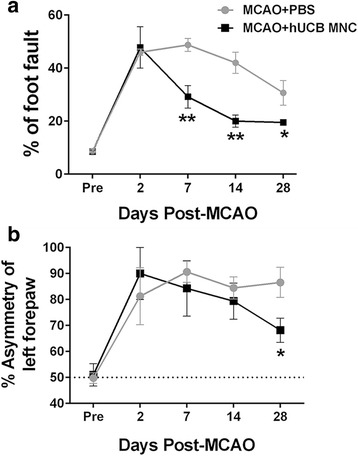
Line graphs of the (**a**) foot-fault test and (**b**) cylinder test of a vehicle- and stem cell-treated animal before middle cerebral arterial occlusion (*MCAO*) (*Pre*), and at 2, 7, 14, and 28 days post-MCAO (*n* = 6 per group; mean ± SEM). *P < 0.05, **P < 0.01, versus the PBS vehicle. *hUCB* human umbilical cord blood, *MNC* mononuclear cell

The foot-fault scores were not significantly different between the vehicle and treatment group before stroke. They increased on day 2 after stroke, followed by some improvement between 7 to 28 days in both groups (Fig. 2a). Improvement was, however, larger and faster in the treatment compared to the vehicle group. The numbers of foot faults were lower in the treatment compared with the vehicle group on days 7, 14, and 28 post-MCAO (29.2 ± 4.3% versus 48.7 ± 2.4% at day 7, *P* = 0.009 by two-way ANOVA with Bonferroni post-hoc test; 20.0 ± 2.3% versus 42.0 ± 4.0% at day 14, *P* = 0.006; 19.5 ± 1.0% versus 30.7 ± 4.7% at day 28, *P* = 0.040), but not on day 2 (*P* = 0.86).

The forelimb asymmetry scores were not statistically different between groups before stroke. They increased 2 to 28 days after stroke in both groups (Fig. 2b). The treatment group showed improvement from day 2 to day 28, reaching significance on day 28 (68.2 ± 4.6% for treatment group versus 86.6 ± 5.8% for PBS-treated group at 28 days, *P* = 0.04 by two-way ANOVA with Bonferroni post-hoc test), whereas the vehicle group showed no improvement from day 2 to day 28. These data show that hyperacute transplantation of hUCB MNCs improved functional recovery at 28 days after stroke.

### hUCB MNC transplantation reduced lesion volume

**Fig. 3:**
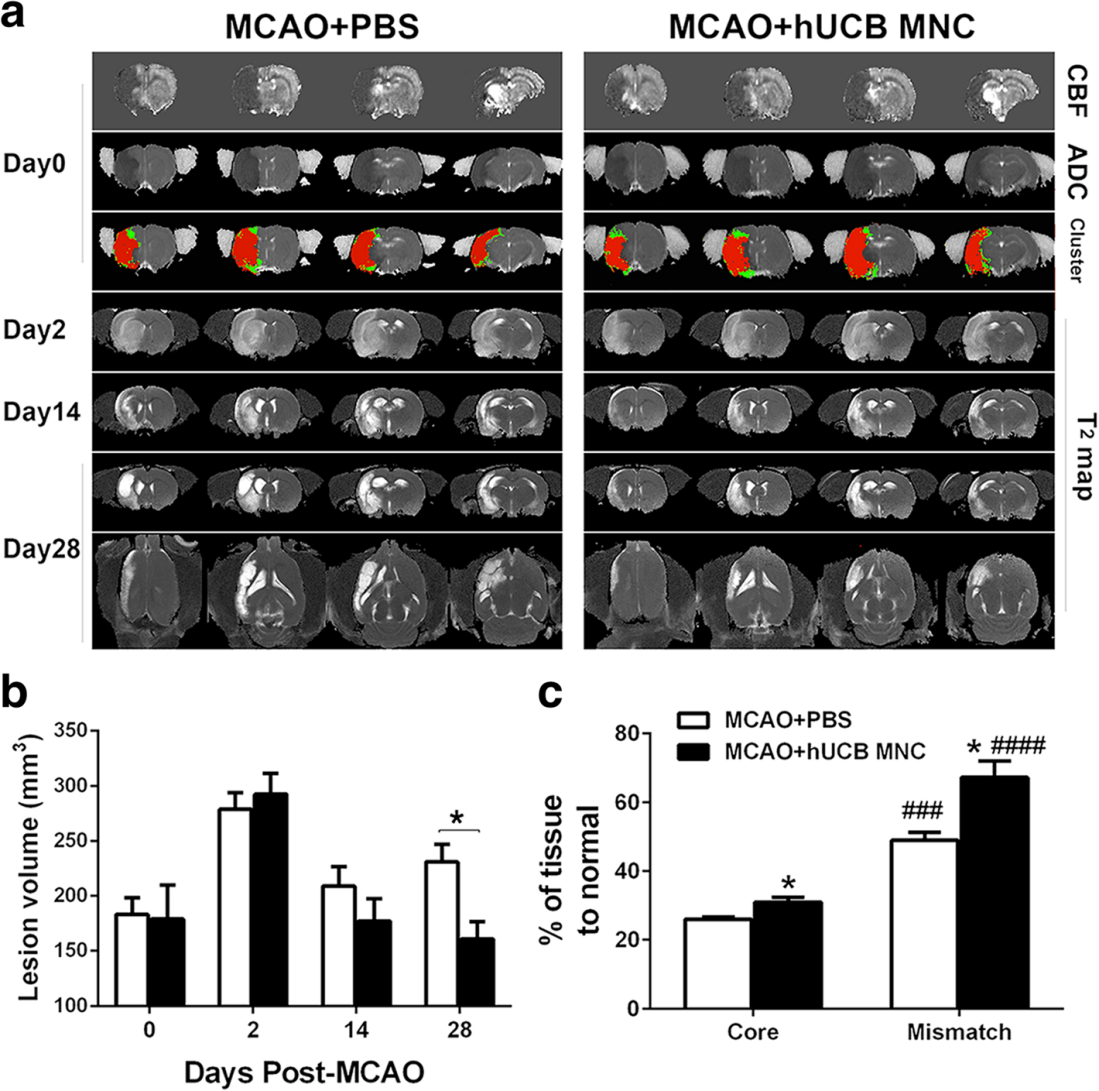
**a** Representative cerebral blood flow (*CBF*), apparent diffusion coefficient (*ADC*), clusters of tissue, and T_2_ map of a PBS vehicle- and stem cell-treated animal at day 0 (30 min), and at 2, 14, and 28 days post-middle cerebral arterial occlusion (*MCAO*). Cluster analysis yielded ‘perfusion-diffusion mismatch’ (*green*), and ‘ischemic core’ (*red*) clusters, based on initial ADC and CBF data. Hypointense areas on the CBF and ADC map show the initial stroke lesion at day 0. Hyperintense areas on the T_2_ map indicate stroke lesion at the following time points. **b** Quantitative analysis of stroke lesion volume over the time course (*n* = 6 per group, mean ± SEM). *P < 0.05. **c** Percentage of rescued core and mismatch tissues were analyzed based on day 28 and day 0 MRI data (*n* = 6 per group, mean ± SEM). *P < 0.05, versus the PBS vehicle; ^###^ *P* < 0.001, ^####^ *P* < 0.0001, versus core tissue of the same group. *hUCB* human umbilical cord blood, *MNC* mononuclear cell, *PBS* phosphate-buffered saline

Ischemic evolution was measured longitudinally by MRI. The initial lesion, determined by ADC at 30 min after MCAO, showed no significant difference between groups (*P* > 0.05 by two-way ANOVA with Bonferroni post-hoc test; Fig. 3a). By comparison, the T_2_ infarct volume at 28 days in the treatment group was significantly smaller than that in the vehicle group (160.9 ± 15.7 versus 231.2 ± 16.0, *P* = 0.04; Fig. 3b).

Analysis was performed to evaluate the treatment effects on the initial (30 min) core and mismatch tissue (Fig. 3c). More initial core and mismatch pixels were rescued in the treatment compared to the vehicle group at 28 days (core: 31.0 ± 1.4% versus 26.0 ± 0.6%, *P* = 0.02; and mismatch: 67.3 ± 4.7% versus 49.0 ± 2.3%, *P* = 0.03 by unpaired *t* test). More mismatch tissue was rescued compared to core tissue in the treatment compared to the vehicle group (*P* < 0.0001).

### hUCB MNC transplantation increased cerebral blood flow in the infarct area

**Fig. 4:**
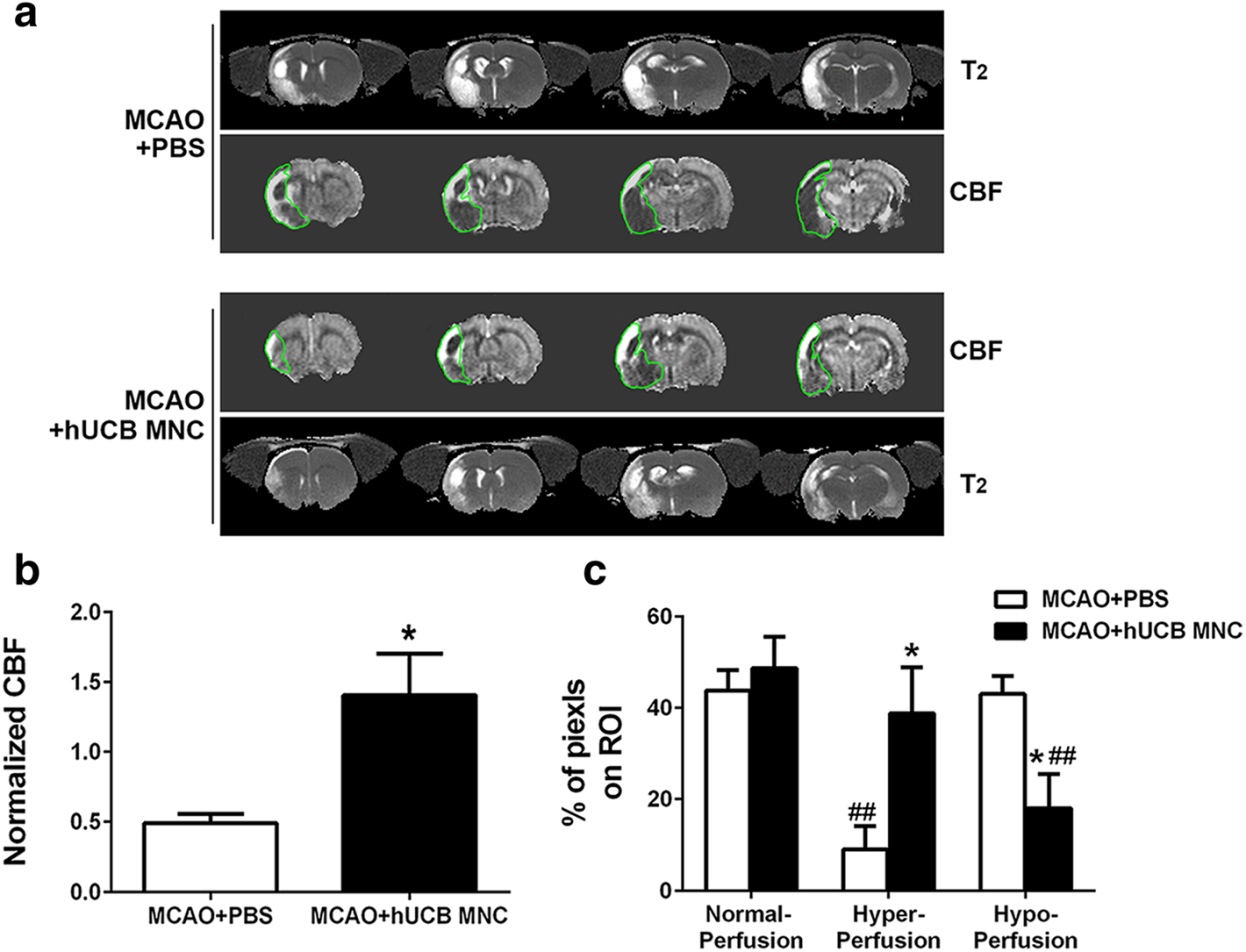
**a** Representative cerebral blood flow (*CBF*) map and corresponding T_2_ map at 28 days post-middle cerebral arterial occlusion (*MCAO*). The region encircled by the *green line* is the region of interest (*ROI*), defined as a T_2_ abnormal area on the corresponding T_2_ map. **b** The CBF value of ROI was quantified. Values were normalized to a homologous region in the contralesional brain. **c** The percentage of pixels (with normal CBF, hyperperfusion, and hypoperfusion) on ROI was calculated. The thresholds for abnormal perfusion were defined as the mean CBF value of the contralateral hemisphere ± 2 standard deviations (*n* = 6 per group, mean ± SEM). *P < 0.05, versus the PBS vehicle; ^##^ *P* < 0.01, versus normal perfusion of the same group. *hUCB* human umbilical cord blood, *MNC* mononuclear cell

CBF in abnormal T_2_ regions (Fig. 4a) was evaluated at 28 days after stroke. Normalized (with respect to the normal hemisphere) CBF values were significantly higher in the treatment compared to the vehicle group (1.41 ± 0.30 versus 0.49 ± 0.07, *P* = 0.04 by unpaired *t* test; Fig. 4b).

We also classified the tissues with different perfusion within the affected area. The treatment group showed more hyperperfusion (38.8 ± 10.2% versus 9.1 ± 5.0%, *P* = 0.02 by two-way ANOVA with Bonferroni post-hoc test) and less hypoperfusion (43.1 ± 3.9% versus 18.0 ± 7.5%, *P* = 0.029) compared to the vehicle group (Fig. 4c).

### hUCB MNC transplantation increased cerebral vascular reactivity

**Fig. 5:**
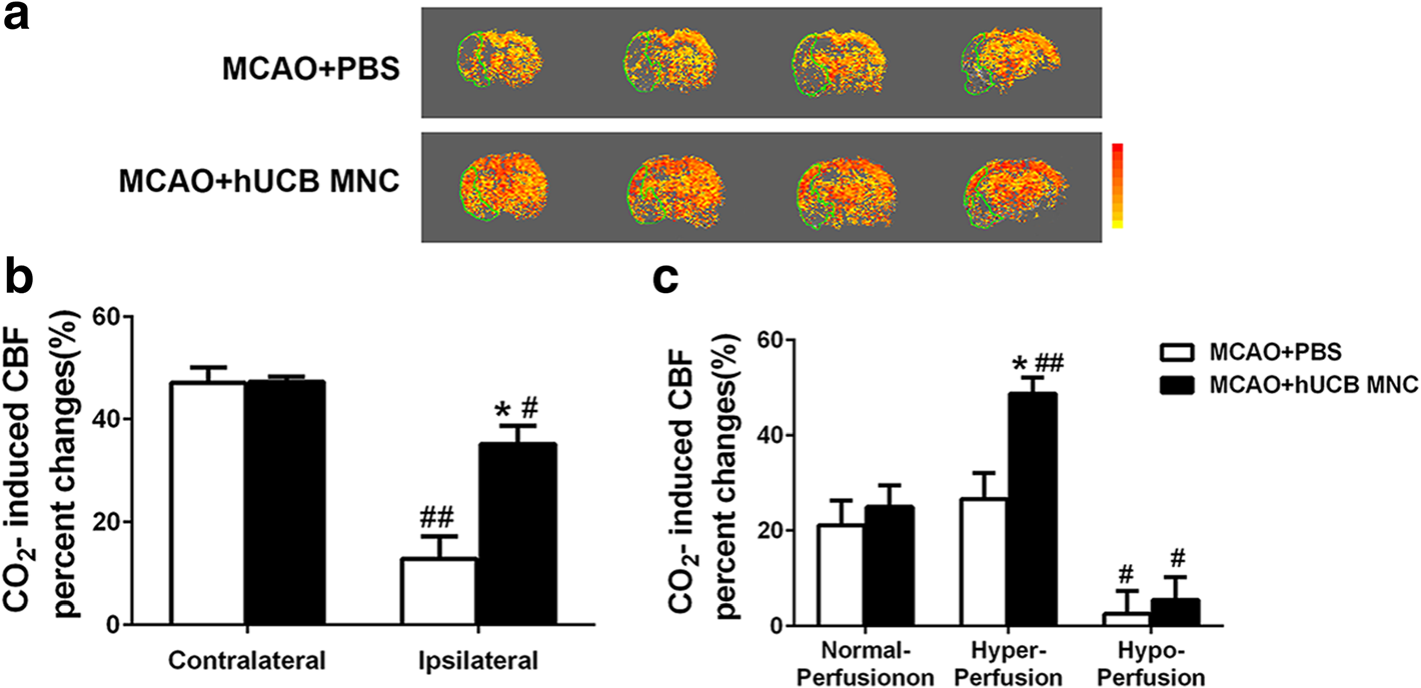
**a** Representative CO_2_ reactivity map from each group at 28 days post-middle cerebral arterial occlusion (*MCAO*). The region encircled by the *green line* is the ROI, defined by the stroke lesion on the corresponding T_2_ map. The color scale bar indicates percentage change in cerebral blood flow (*CBF*) ranging from 1% to 100% (*yellow–red*). **b** Quantification of percentage CBF changes responding to 5% CO_2_ challenge from ROI (*n* = 6 per group, mean ± SEM). *P < 0.05, versus the PBS vehicle; ^#^ *P* < 0.05, ^##^ *P* < 0.01, versus contralateral homologous regions. **c** CO_2_ induced percentage change in CBF were analyzed separately on normal, hyper-, and hypoperfusion areas of ROI (*n* = 6 per group, mean ± SEM). *P < 0.05, versus the PBS vehicle; ^#^ *P* < 0.05, ^##^ *P* < 0.01, versus normal perfusion of the same group. *hUCB* human umbilical cord blood, *MNC* mononuclear cell

Cerebrovascular reactivity was evaluated using CBF fMRI at 28 days post-MCAO (Fig. 5a). Compared to the contralateral normal hemisphere, CO_2_-induced CBF percentage changes were lower in the ipsilateral hemisphere in both groups (12.8 ± 4.3% versus 47.1 ± 3.1%, *P* = 0.001 by unpaired *t* test in the vehicle group; 35.2 ± 3.5% versus 47.3 ± 1.0%, *P* = 0.012 in the treatment group). However, CBF percentage changes within the infarcted area were significantly higher in the treatment compared to the vehicle group (35.2 ± 3.5% versus 12.8 ± 4.3%, *P* = 0.016; Fig. 5b). Further analysis showed that CO_2_-induced CBF increases were larger in hyperperfused tissue compared to those in the normal and hypoperfused tissues. Such CBF changes in the hyperperfusion area were more dramatic in the treatment compared to the vehicle group (48.7 ± 3.4% versus 26.6 ± 5.5%, *P* = 0.027 by two-way ANOVA with Bonferroni post-hoc test; Fig. 5c). CO_2_-induced CBF percentage changes in the hyperperfusion area were higher than those in the normal perfusion area in the treatment group (48.7 ± 3.4% versus 25.0 ± 4.6%, *P* = 0.009).

### hUCB MNC treatment promoted vascular remodeling after stroke

**Fig. 6:**
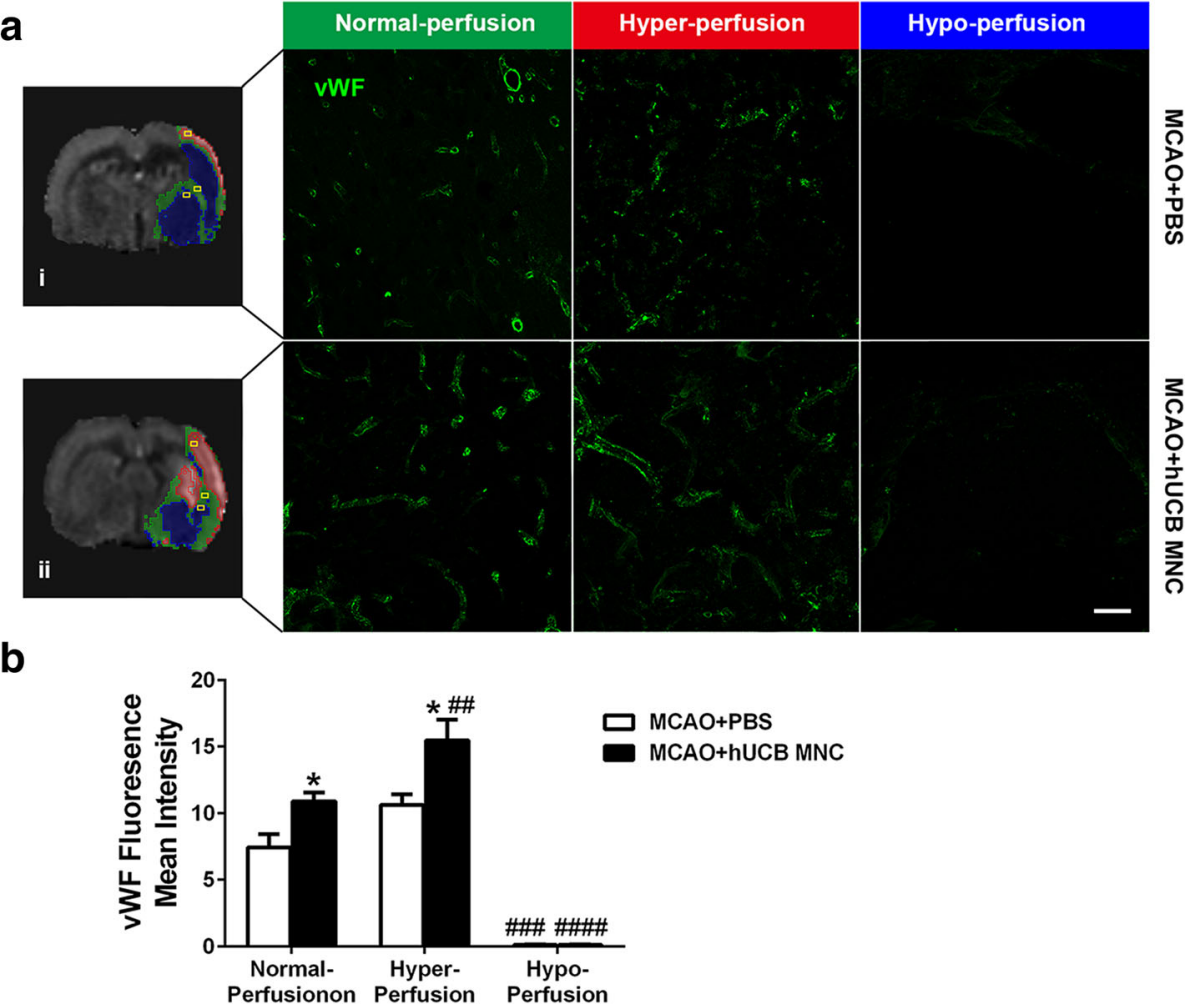
Vascular remodeling was evaluated by von Willebrand factor (*vWF*) immunostaining. **a** Representative perfusion MRI map showing normal, hyper-, and hypoperfusion areas in (i) the vehicle group and (ii) the treatment group at 28 days after stroke. The *yellow frame* presents the field of view selected in the analysis of vWF immunostaining. Higher magnification images of vWF staining are shown in correlated normal CBF, hyperperfusion, and hypoperfusions areas of the two groups. *Scale bar* = 100 μm. **b** Mean intensity of vWF fluorescence in ROI were quantified (*n* = 6, mean ± SEM). *P < 0.05, versus the PBS vehicle; ^##^ *P* < 0.01, ^###^ *P* < 0.001, ^####^ *P* < 0.0001, versus normal perfusion of same group. *hUCB* human umbilical cord blood, *MNC* mononuclear cell

To explore possible mechanisms of regional CBF and vascular reactivity improvements by hUCB MNC treatment, vascular morphology and density of tissues with different CBF was analyzed using vWF immunofluorescent staining (Fig. 6a). vWF staining showed a more defined vascular morphological shape in the treatment group. The mean intensity of vWF fluorescence was significantly higher in the tissue with normal perfusion and hyperperfusion in the treatment group compared to the vehicle group (normal perfusion: 10.9 ± 0.7 versus 7.4 ± 1.0, *P* = 0.031; hyperperfusion: 15.5 ± 1.6 versus 10.6 ± 0.8, *P* = 0.034 by two-way ANOVA with Bonferroni post-hoc test; Fig. 6b). Moreover, intensity of vWF from the hyperperfusion area was higher than intensity from the normal perfusion area in the treatment group (15.5 ± 1.6 versus 10.9 ± 0.7, *P* = 0.008). These findings suggest hUCB MNC transplantation enhanced vascular density in the infarcted area during stroke recovery.

### Engrafted hUCB MNC participated in vasculogenesis after transplantation

**Fig. 7:**
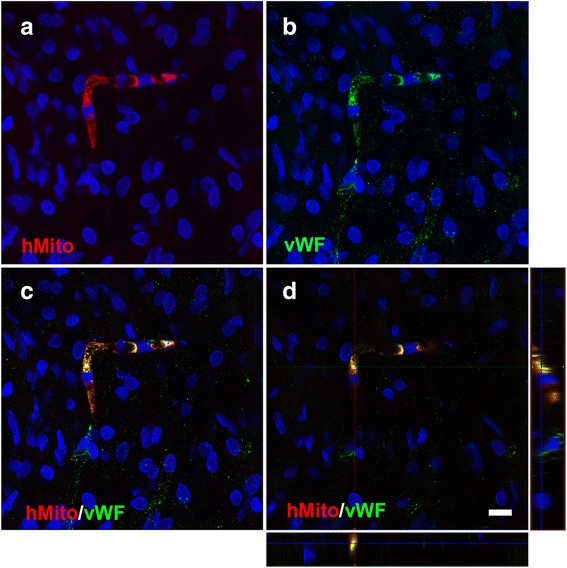
**a** Human mitochondrial (*hMito*) immunofluorescent staining. **b** The vascular endothelial marker von Willebrand factor (*vWF*) immunofluorescent staining. **c** Double staining and (**d**) orthographic projection show co-localization of hMito with vWF on brain slices at 28 days post-MCAO. DAPI (*blue*) staining was used to identify nuclei. *Scale bar* = 10 μm

To evaluate whether transplanted cells participate in vasculogenesis, double-immunolabeling of the human cell marker human mitochondrai (hMito) and vWF were used (Fig. 7). hMito-positive engrafted hUCB MNCs were detected in the ipsilesional cortex at 28 days after transplantation. The hMito-positive engrafted hUCB MNCs appeared as microvascular-like structures and stained positive for vWF, indicating engrafted cells had differentiated into endothelial cells. In addition, some engrafted cells made connections with host endothelial cells (the latter were vWF-positive but hMito-negative), indicating engrafted cells interacted with the host vasculature.

## Discussion

This study provides evidence that intraarterial transplantation of human umbilical cord blood mononuclear cells in the hyperacute phase of ischemic stroke improves regional CBF, cerebrovascular reactivity, and vascular remodeling in which transplanted cells were found to participate in vasculogenesis. Hyperacute stem cell transplantation also reduced behavioral deficits and infarct volume. A novelty of this study is that multimodal MRI was used to track ischemic evolution, verify “patient selection”, and evaluate the effects of treatment on different tissue types associated with stem cell transplantation, with corroboration by immunohistology and behavioral function. This study is the first to report the effect of hyperacute intraarterial transplantation of human UCB cells on CBF and vasoreactivity in vivo.

Human umbilical cord blood mononuclear cells (hUCB MNCs) comprise multiple stem/progenitor cells, including HSCs, MSCs, EPCs, and so forth. Many preclinical studies have shown the efficacy of hUCB MNCs for treating stroke, either as hUCB MNCs or separate cell types (e.g., MSCs, EPCs). For example, Boltze et. al. [9] showed that intravenous infusion of hUCB MNCs less than 72 h after stroke improved recovery of behavioral function. Also, intravenous injection of AC133+ EPCs derived from hUCB cells reduced the infarct volume of stroke rats [27], while a recent study shows that the therapeutic effect of hUCB MNC intraarterial transplantation is better than hUCB-derived MSCs alone [5].

### Effects on lesion volume, mismatch evolution, and behavioral function

Selection of intraarterial rather than intravenous injection was based on the evidence that more engrafted cells reach the stroke lesion when transplanted intraarterially [28]. We opted for a dose of 5 × 10^6^ cells for intraarterial injection based on a dose-dependent study that shows intravenous injection of over 10^6^ cells 1 day after stroke was sufficient to improve behavioral and histopathologic recovery for cord blood cell transplantation [10]. However, further study is needed to optimize the dosage for intraarterial transplantation in the hyperacute phase of ischemic stroke.

We found hyperacute intraarterial transplantation of hUCB MNCs salvaged more initial diffusion-perfusion mismatch and ischemic core tissue, and reduced infarct volume 28 days after stroke. This approach provides information about the tissue types that are affected by the treatment in a longitudinal fashion, which offers an advantage over terminal histological approaches to evaluate infarct volumes. Also, we used MRI to strictly select ‘patients’ in this study, which could reduce the variability of experiments and minimize the usage of animals. The improvement in infarct volumes is corroborated by improvement in behavioral scores in the treatment group at 7, 14, and 28 days after stroke. While both foot fault and forelimb asymmetry scores showed improvement in the treatment compared to the vehicle group, there were differences in temporal patterns. For example, in the vehicle group, the foot fault scores improved with time but the forelimb asymmetry scores did not. The difference between groups was smaller in the forelimb asymmetry score on days 7 to 14. A likely explanation is that the forelimb placement task is less challenging than the foot-fault task, allowing the animals to readily compensate for the deficits; thus, smaller group differences were observed in the forelimb placement task. The same improving trend as for the foot-fault test was found for the cylinder test at 7 and 14 days, even though there was no significant differences between the two groups. This is why multiple behavioral tests were included in the study; the sensitivity of tests differ over time. Reduced behavioral deficits by intraarterial transplantation of hUCB MNCs 24 h after stroke have been reported up to 14 days after stroke [5]. Surprisingly, there was no significant difference on the infarct volume between the two groups at the early time points (day 2) after stroke. However, a similar result was reported by Zhu et al. [29] where hUCB MSCs were delivered via intracerebral injection 24 h after MCAO. The transplantation did not significantly reduce infarct volume at 3 days post-stroke, but it improved functional recovery in mice 14 days post-stroke [29]. This finding likely reflects the therapeutic effect of acute hUCB MNC treatment on improving brain recovery rather than neuroprotection at the acute phase. Based on this, and the nature of cord blood cells, we next focused on the impact of cell transplantation on the cerebrovascular function at the chronic phase of stroke.

### Effects on cerebral vasculature

We found hUCB MNC transplantation increased CBF to the stroke-affected region. Improved perfusion after stroke has been associated with improved functional recovery [30], and this may partly explain the reason for functional improvement in our stem cell-treated group. Furthermore, normalization of CBF improves the delivery of nutrients and oxygen to support brain tissue in the injured area, minimizing neuronal cell death and promoting brain recovery [31, 32]. Similarly, a tendency for CBF increase to the stroke-affected hemisphere has been observed at 14 days post-stroke following intravenous injection of hUCB-derived AC133+ EPCs 24 h after MCAO, even though this trend did not reach significance [27]. Meanwhile, intracerebral transplantation of hUC-derived MSCs has been shown to increase local CBF in the ischemic hemisphere in stroke rats as measured by laser Doppler flowmetry [33]. MRI as used in our study, in contrast to laser Doppler flowmetry, enables CBF measurements beyond the cortical surface and correlation with perfusion-diffusion mismatch. Furthermore, our results indicate that less tissue suffered hypoperfusion and more tissues experienced hyperperfusion in the stem cell-treated group. A previous study has also demonstrated increased regional hyperperfusion during stroke recovery that may be related to angiogenesis [34].

We also found hUCB MNC transplantation significantly improved cerebral vasoreactivity in the stroke-affected hemisphere. Cerebrovascular reactivity has been showed to be attenuated for an extended period following ischemic injury [35], and improvement in cerebrovascular reactivity has been associated with recovery after stroke [14, 15, 36]. Our findings demonstrate that hUCB MNC treatment has beneficial effects on vascular function in general and likely contributes to stroke recovery and functional improvement at 28 days. It is worth noting that vascular function has been shown to be correlated with stroke recovery [15, 37], and functional MRI is a unique and powerful tool to longitudinally monitor cerebral vascular function in vivo [38]. To our knowledge, this is the first report that early intraarterial transplantation of hUCB MNCs in vivo improves cerebrovascular reactivity in chronic stroke.

To corroborate MRI findings of basal CBF and vasoreactivity improvement, we performed histological experiments in which different ischemic tissue types were chosen for histology based on multimodal MRI. We found more intact vascular morphology and increased vascular density in tissue with hyperperfusion and normal perfusion in the treatment group compared to the vehicle group, suggesting enhanced angiogenesis and vascular remodeling. These findings are consistent with previous reports of enhanced angiogenesis and vascular remodeling after intravenous or intracortical transplantation of UCB cells [33, 39, 40]. The vascular density in tissues with hyperperfusion was higher than that in tissues with normal perfusion, suggesting increased vascular density is correlated with hyperperfusion in subchronic stroke [34, 41].

This improvement is at least in part due to the engrafted hUCB MNCs forming new vessels during stroke recovery, as indicated by the engrafted cells showing microvascular-like structure and expressing the endothelial marker vWF. Moreover, we also found some engrafted cells made connections with host endothelial cells. hUCB cells have been shown to differentiate into endothelial cells and survive for an extended period after transplantation [4, 33, 42]. In addition, engrafted cells could also secrete growth factors, such as angiogenic factors (e.g., vascular endothelial growth factor [43]), which could further contribute to enhanced angiogenesis. Although further mechanistic studies are needed, these findings together suggest hUCB MNCs transplanted early after stroke participate in improving vascular function during recovery.

### Limitations of the studies and future directions

We chose 60-min MCAO as this duration yielded a reasonable volume of initial ischemic lesion and diffusion-perfusion mismatch under our experimental conditions [19, 23]. However, different durations of occlusion will need to be explored to mimic various clinical conditions of ischemic stroke. This study used male, healthy young adult rats. Future studies will need to use older animals of both genders and with comorbidities. While we showed that intraarterial hUCB MNC delivery is effective, studies are need to investigate whether hyperacute intravenous cell delivery has a similar effect on vascular function. We chose cell infusion immediately after reperfusion to test the therapeutic potential of combination treatment (thrombectomy and stem cell infusion). Further work is needed to explore the optimized time window of HUCB MNC intra-arterial infusion. Although endothelial cell staining using its specific marker (e.g., vWF, CD31) is the widely accepted and used method to study vascular density [25, 27], intravenous perfusion with fluorescent-labeled lectin could show better spatial resolution and connection of the vascular network.

Mechanical thrombectomy is becoming a promising treatment for stroke patients with intracranial large artery occlusions presenting within 6 h of symptom onset [16]. The time from stroke onset to arterial recanalization significantly impacts outcomes [44], prompting discussion on reorganizing the stroke system of care to facilitate rapid reperfusion and improve prompt access of these therapies to patients in need [45]. Intraarterial infusion of UCB MNCs immediately after mechanical thrombectomy could be a potential adjuvant therapy. The demonstrated improvement in cerebrovascular function is particularly relevant for hyperacute treatment. The allogeneic nature of UCB MNCs could be a practical option for use as an ‘off-the-shelf’ product readily available for therapeutic use.

## Conclusions

Intraarterial human UCB MNC infusion administered in the hyperacute stroke phase improves regional CBF, cerebrovascular reactivity, and vascular function, while reducing behavioral deficits and infarct volume. The engrafted UCB MNCs differentiate into endothelial cells and interact with the host vasculature. Hyperacute intraarterial infusion of UCB MNCs could serve as a potential adjuvant therapy for post-mechanical thrombectomy.

## Abbreviations

ADC: Apparent diffusion coefficient
cASL: Continuous arterial spin labeling
CBF: Cerebral blood flow
DAPI: 4’,6’-Diamidino-2-phenylindole
ECA: External carotid artery
EPC: Endothelial progenitor cell
fMRI: Functional magnetic resonance imaging
hMito: Human mitochondria
HSC: Hematopoietic stem cell
hUCB: Human umbilical cord blood
ICA: Internal carotid artery
MCAO: Middle cerebral arterial occlusion
MNC: Mononuclear cell
MRI: Magnetic resonance imaging
MSC: Mesenchymal stem cell
PBS: Phosphate-buffered saline
PFA: Paraformaldehyde
ROI: Region of interest
UCB: Umbilical cord blood
vWF: von Willebrand factor
